# Genome sequence of pineapple secovirus B, a second sadwavirus reported infecting Ananas comosus

**DOI:** 10.1007/s00705-022-05590-9

**Published:** 2022-10-21

**Authors:** Adriana Larrea-Sarmiento, Andrew D.W. Geering, Alejandro Olmedo-Velarde, Xupeng Wang, Wayne Borth, Tracie K Matsumoto, Jon Y Suzuki, Marisa M Wall, Michael Melzer, Richard Moyle, Murray Sharman, John Hu, John E. Thomas

**Affiliations:** 1 Department of Plant and Environmental Protection Sciences, University of Hawaii, Honolulu, HI USA; 2 Queensland Alliance for Agriculture and Food Innovation, Centre for Horticultural Science, The University of Queensland, Ecosciences Precinct, 4001 Brisbane, QLD GPO Box 267, Australia; 3 Department of Agriculture, Agricultural Research Service, United States, Daniel K. Inouye U. S. Pacific Basin Agricultural Research Center, Hilo, HI USA; 4 School of Agriculture and Food Sciences, The University of Queensland, 4072 St Lucia, QLD Australia; 5 Department of Agriculture and Fisheries, Ecosciences Precinct, 4001 Brisbane, QLD GPO Box 267, Australia

## Abstract

The complete genome sequence of pineapple secovirus B (PSV-B), a new virus infecting pineapple (*Ananas comosus*) on the island of Oahu, Hawaii, was determined by high-throughput sequencing (HTS). The genome comprises two RNAs that are 5,956 and 3,808 nt long, excluding the 3’-end poly-A tails, both coding for a single large polyprotein. The RNA1 polyprotein contains five conserved domains associated with replication, while the RNA2 polyprotein is cleaved into the movement protein and coat protein. PSV-B is representative of a new species in the subgenus *Cholivirus* (genus *Sadwavirus*; family *Secoviridae*), as the level of amino acid sequence identity to recognized members of this subgenus in the Pro-Pol and coat protein regions is below currently valid species demarcation thresholds.

**Supplementary Information:**

The online version contains supplementary material available at 10.1007/s00705-022-05590-9.

Plant viruses of the family *Secoviridae* (order *Picornavirales*) mainly infect dicotyledonous plant species and are naturally transmitted by nematodes or arthropods. The capsids of these viruses are icosahedral, 25 to 30 nm in diameter, and are composed of 60 coat protein (CP) subunits [10, 11]. The secovirus genome can be monopartite or bipartite. Bipartite genomes are divided between two RNA segments (RNA1 and 2), each modified by the addition of a 3’-terminal poly(A) tail and a covalently bound genome-linked viral protein (VPg) at the 5’end. RNA1 ranges in length from 6 to 8 kb and encodes the proteins necessary for cytoplasmatic replication. In contrast, RNA2 is smaller, ranging from 2 to 4 kb, and encodes the movement protein (MP) and up to three CPs [8]. The Pro-Pol sequence on RNA1 is used to infer phylogenetic relationships within the family and to other members of the order [8]. Currently, members of the family *Secoviridae* are classified into nine genera: *Comovirus*, *Fabavirus*, *Nepovirus*, *Cheravirus*, *Sadwavirus*, *Torradovirus*, *Sequivirus*, *Stralarivirus*, and *Waikavirus* [8, 11]. In addition, the genus *Sadwavirus* is divided into three subgenera, *Stramovirus*, *Satsumavirus*, and *Cholivirus* [8].

Pineapple secovirus A (PSV-A), a recently assigned member of the family *Secoviridae*, was first detected in pineapple (*Ananas comosus*) from the germplasm accession HANA 187 from the Pacific Basin Agricultural Research center (PBARC) in Hilo, Hawaii [5]. A PSV-A survey was carried out in 2019 on the island of Oahu and showed the presence of the virus in six out of twelve plants with symptoms of reddening and wilting of leaves associated with mealybug wilt of pineapple (MWP). PSV-A was absent in the 13 asymptomatic plants tested [5]. Various combinations of ampeloviruses from the pineapple mealybug wilt-associated virus (PMWaV) species complex were also detected in the 12 symptomatic plants (Larrea-Sarmiento et al, unpublished results).

To examine the presence of further undiscovered viruses infecting *A. comosus*, high-throughput sequencing (HTS) was performed on the same field plants detailed in a study by Larrea-Sarmiento et al. [5]. Total RNA was extracted from the basal portions of individual pineapple leaf samples using a Spectrum™ Total RNA Kit (Sigma-Aldrich, USA), following the manufacturer’s instructions. Total RNA extracted from the 12 MWP-symptomatic field samples and 13 healthy-looking plants were pooled into two respective composite RNA samples and subjected to ribodepletion to remove the ribosomal RNA (rRNA). cDNA library synthesis was followed by HTS using an Illumina® NovaSeq 6000 system to obtain paired-end reads (2 × 100 bp) at the Genomics High-Throughput Sequencing Facility at the University of California, Irvine.

Data obtained from ~ 40million raw reads per composite ribosomal RNA-depleted total RNA were curated and assembled following the methods of Green et al. [2]. The resulting contigs were annotated by doing BLASTx searches of the NCBI virus sequence database. Annotated contigs revealed sequence similarity to the previously characterized PMWaVs and secoviruses. Two contigs recovered from the symptomatic composite sample dataset had significant matches to PSV-A and other sadwaviruses but were sufficiently divergent to suggest that they represented the two RNA components of a new virus. Similar to PSV-A and the majority of secovirids, the potential new virus has a bipartite genome consisting of two positive-sense RNA molecules.

To obtain the complete genome sequence of the virus, 5’ and 3’ rapid amplification of cDNA ends (RACE) was performed. Both 5’ and 3’ ends were obtained using a Takara SMARTer RACE 5’/3’ Kit according to the manufacturer’s instructions, followed by PCR with a universal anchored primer and sequence-specific primers (Supplementary Table S1). Amplicons were cloned, and five to seven clones were sequenced by the Sanger method. The complete genome comprises two RNA molecules; RNA1 is 5,956 nt long (GenBank accession no. OM777135) and RNA2 is 3,808 nt long (GenBank accession no. OM777136), each coding for large polyproteins referred as P1 and P2, respectively. The name "pineapple secovirus B" (PSV-B) is proposed for this putative new virus infecting pineapple.

The polyprotein precursor P1 of PSV-B is 1,875 aa long and is composed of proteins involved in replication: protease cofactor (Pro-C), helicase (Hel), VPg, protease (Pro), and RNA-dependent RNA-polymerase (Pol). Likewise, the polyprotein P2 of PSV-B is 1,143 aa long and is composed of a movement protein (MP) and one large coat protein (CP) (Fig.1). Similar to other secovirids, both PSV-B RNAs are expected to possess a VPg bound at the 5’end and a poly(A) tail at the 3’end, respectively [1, 8]. Analysis of conserved domains using the NCBI Conserved Domain Search Tool (https://www.ncbi.nlm.nih.gov/Structure/cdd/wrpsb.cgi) showed the presence of conserved motifs in the Pol and Hel of P1, whereas a conserved MP motif was predicted for P2. Parallel analysis using HMMER (https://www.ebi.ac.uk/Tools/hmmer/) and Pfam (http://pfam.xfam.org/) further predicted the presence of two CP domains similar to the nepovirus and picornavirus CP domains of P2, although other members within the subgenus *Cholivirus* (genus *Sadwavirus*) are predicted to encode only one large CP [4, 5, 7, 12]. Analysis of the predicted cleavage sites located four Q/S and five E/G dipeptides in the polyprotein P1 [3]. The cleavage sites recognized by the RNA1-encoded 3C-like protease (3CL-Pro) likely cleave P1 at four sites, defining five domains, while 3CL-Pro likely cleaves P2 at one site, defining two domains (Fig.1) [1, 7, 8].

**Fig. 1:**
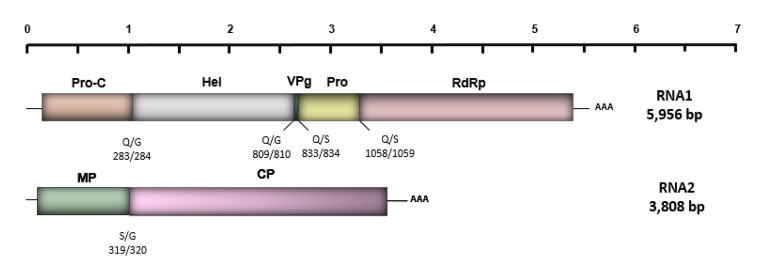
Genome organization of pineapple secovirus B (PSV-B). Predicted cleavage sites with their corresponding dipeptide in the P1 and P2 polyproteins are shown as vertical lines under each RNA segment of PSV-B. Numbers indicates the amino acid position. RNA1 (5,956 nt) encodes a large polyprotein predicted to be cleaved into five proteins: protease cofactor (Pro-C), helicase (Hel), genome-linked viral protein (VPg), protease (Pro), and RNA-dependent RNA polymerase (Pol). RNA2 (3,808 nt) is predicted to code for the movement protein (MP) and one large coat protein (CP). Q, glutamine; G, glycine; S, serine. “AAA” at the 3’ end position of each RNA segment represents the poly-A tail.

The recently characterized PSV-A was found to be closely related to Dioscorea mosaic associated virus (DMaV) and chocolate lily virus A (CLVA) [5]. In 2020, the proposed revision of the family *Secoviridae* classified DMaV and CLVA, previously denoted as unassigned secoviruses, as members of the subgenus *Cholivirus* within the genus *Sadwavirus* [8]. To study the taxonomic position of PSV-B and its relatedness to PSV-A and other members of the family *Secoviridae*, phylogenetic analysis using the maximum-likelihood method based on the aa sequence of the Pro-Pol region was carried out using LG (Le Gascuel) + G (discrete Gamma distribution) as the best model of protein evolution. This analysis suggested that PSV-B is a new *Sadwavirus* member that is related to, but distinct from, the previously characterized *Cholivirus* member PSV-A (Fig.2). PSV-B is placed on a branch distinct from PSV-A and a basal clade that contains DMaV and CLVA (Fig.2). For members within the family *Secoviridae*, the species demarcation criteria are < 80% identity for the aa sequence of the Pro-Pol region and < 75% identity for the large and small CP together [11]. Sequence identities of 45.1% and 53.5% were observed when comparing the Pro-Pol region of PSV-B to PSV-A and CLVA homolog regions, respectively, using pairwise comparisons. Likewise, amino acid sequence identity values of 23.5% and 25.4% were obtained when comparing the CPs of PSV-B and PSV-A and those of PSV-B and CLVA, respectively. These results are consistent with the findings reported in Australia in 2002, where two isometric viruses were described infecting pineapple and partial sequences showed similarities to strawberry mottle virus [9]. All of these results suggest that PSV-B is a member of a new species in the secovirid subgenus *Cholivirus*.

**Fig. 2:**
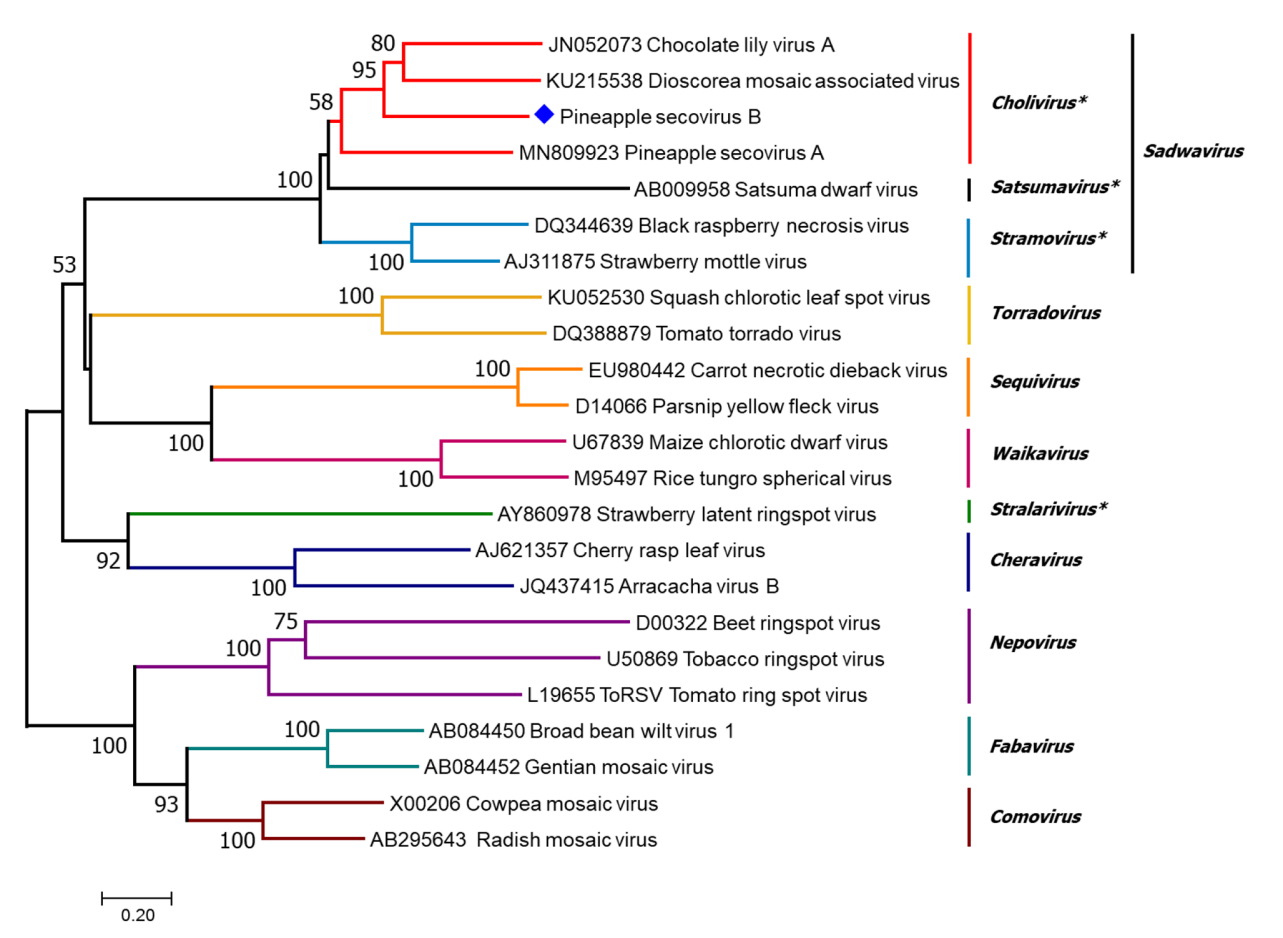
Phylogenetic relationships of the Pro-Pol region of pineapple secovirus B (PSV-B) to other members of the family *Secoviridae*. The maximum-likelihood method with the LG + G matrix-based model was used with 1,000 bootstrap pseudoreplicates as percentage values for branch support. Predicted amino acid sequences were used, and the respective GenBank accession number is shown with each virus name. The alignment was generated using Clustal and implemented in MEGA v.7.0.1. Asterisks represent the three subgenera within the genus *Sadwavirus* and the recently established genus *Stralarivirus.* The blue diamond indicates PSV-B

Two PSV-B-specific primers sets designed based on genomic RNAs 1 and 2 (Supplementary Table S1) were used to test for this virus in 25 field samples collected in 2019. Four out of the 12 MWP symptomatic samples yielded the expected bands of 702 bp for RNA1 and 380 bp for RNA2, and their identity was confirmed by direct Sanger sequencing of the amplicons. The presence of PMWaVs and PSV-A was assessed as reported previously [2, 5, 6]. Samples infected with PSV-B were also infected with PMWaV-2, PMWaV-3, PMWaV-6, and PSV-A. None of the 13 healthy-looking plants were found to be infected by PVS-B. Further research is needed to determine the prevalence of PSV-B in other pineapple-producing countries and to evaluate if it is involved in the etiology of MWP.

## Electronic Supplementary Material

Below is the link to the electronic supplementary material

Supplementary Material 1

Supplementary Material 2
