# Supplementary material for: Genome sequence of pineapple secovirus B, a second sadwavirus reported infecting Ananas comosus
Source: Arch Virol. 2022 Oct 21;167(12):2801–4. doi: 10.1007/s00705-022-05590-9 (PMC9741570; doi:10.1007/s00705-022-05590-9)
Supplement: Supplementary file 2 — Supplementary Material 2 [file 705_2022_5590_MOESM2_ESM.docx]

**Annotated Sequence Records**

**Archives of Virology**

**Genome sequence of pineapple secovirus B, a second sadwavirus reported infecting *Ananas comosus***

Adriana Larrea-Sarmiento^1^, Andrew D.W. Geering^2^, Alejandro Olmedo-Velarde^1^, Xupeng Wang^1^, Wayne Borth^1^, Tracie K Matsumoto^3^, Jon Y Suzuki^3^, Marisa M Wall^3^, Michael Melzer^1^, Richard Moyle^4^, Murray Sharman^5^, John Hu^1*^, John E. Thomas^2*^

^1^ Department of Plant and Environmental Protection Sciences, University of Hawaii, Honolulu, HI, USA

^2^ The University of Queensland, Queensland Alliance for Agriculture and Food Innovation, Centre for Horticultural Science, Ecosciences Precinct, GPO Box 267, Brisbane, QLD 4001, Australia.

^3^ United States Department of Agriculture, Agricultural Research Service, Daniel K. Inouye U. S. Pacific Basin Agricultural Research Center, Hilo, HI, USA.

^4^The University of Queensland, School of Agriculture and Food Sciences, St Lucia, QLD 4072, Australia.

^5^Department of Agriculture and Fisheries, Ecosciences Precinct, GPO Box 267, Brisbane, QLD 4001, Australia.

^*^ Corresponding authors: [johnhu@hawaii.edu](mailto:johnhu@hawaii.edu), [j.thomas2@uq.edu.au](mailto:j.thomas2@uq.edu.au)

**PSV-B_RNA1**

ACATGGGAAACAAAGCGAACGTTTCTCTTCAGCGACCGTCACTTTCCTTGCGAACTTAATTGCGATCTTTCTTTTGATTGTCTCTTCGACTTGTTCTACTTCGTTTCTTGCTTAGCAAATCTGACCTGACCCTATTTAGGGCGCTAAACAGGCTCCAGTCGTATCGTAACGATCGTTTCATACCACTTCAATGGCCTCTCAGGTTGAAAGCGATTGCTTACGCTATGTTGTTACAGAAGAGGAAGCCCTCCTCGTTTTAGAGGCAGACTTCCTCATTTGCTATTTTGAGGAACTTGAACGCACTGGTAACGTGTGCGTGCCAGCTCCTGCTGGTGGCTCAGCTGATAATGATATCCGTCAATCAGTGATGGAAATTGTTAATAAATTACCAGCTGAACACCCTGCCTCCACCGACGTCCAACTGGCAGAATGTCTATTCTTCGATGTTGGTGAGGATCTGAGGAAAGGATTGGACAAGCATTTCTATCCTCATTTCGATAGAAATGATGCTGACTATAATCCACAAGGCATCCTCGGCAGTTTTGCTCAAGGTGCCTTCATAGAAATTGGTAGTCGCACTATTGGTCGTCTGATGGCCAAAGTGGGCTCAGCTGTGAGTCCTTTTTCTCAACTACTCGCTAAAATGGATATCATTTTAGACAAAGTAGTTAGTGCTTTTGATTGGCTTGCAAATATCGTTGATGGATGCATGAATTTTCTGACCTCACTTAGGGACAAATTCAAATCACTGATTCAGACTTGTCTTGAGAAACTTAAATGCTTTGCTGAACACTTTAGCTACTTAATGCCGTTAGTTTGCGGAGTGTTCTTTTCATCATGTTTCTTTTTTCTATTGAATAAATTTTTGAGCTATGTCGCGCCTTCATATGTTATGTCTTATGGAAGGTTGGTTGAAATAATATCAATTGTTGCAGCAGTCATTGGAATAAAGGAATTTGGTCAATACTTTATGACCATGGAATCTGATGCTCGGAAGTCCTTCATAAACGCTATCAAGAACTTTCTGGGCTTTGGTGATGTTGAAAGTGGCTTGGCTGTGAATGAAAGTGAGCCACAAGTACAATCAGGCCTCATGGACGTTGGACTGTTTGGTTGCCTTGTCTCCATGATTACATTCTTTGCAGATGAAAAGTTTCGGGTCGATTTCTGGGGATTTGCAAAAACTGCAGCGGCTCTGAAGAATATCTCAGATGGATACGACAAAGTTAGTAAGATGATGGGAGACATCACTCTATGGTTCTTCCAAAAACTTGGTACGAGTGGTATCGATGGAAGTGGTGCTGCACAAGCTCTCATGTTGCAAACTGGGATTGGGCTTAGTCAGTGGATGGAAGATTGTGAAAAACTCATAATAGAGGGCAACACGGTCACCCATTCAATGCAGTGGGTCTTTTCTGAGAGTCGGCGCCTAATTGATCAAGGAGCCGTCATTTCTAGTTATTTTGCTAGAACTAATGACGGCACGACTTTCCACTTGCGAGCGCGATTCGTGGCTGTGGAGAAGAGCCTAAAAGAATTTTATGGGAAAATCAAACAGGCTAATCTGAGTAATCAACATAGACTGACTCCTTTCACAATTTGCTTCATGAGTGAACCTGGAGTTGGAAAATCAACAGCTGTACGACCCTTTTGCGACACCTTCCTAGATGCTATGGGTGAACCCAAAGCTGATAGGATTTATACAAGAAATGGCGGGGACGCGTACTGGTCGAACTACATACGTCAGCCGTGTGTTTTGTTTGACGATTTTGGACAGACGCGCCAAGAAAATAATAGGTTTGATGAGGAAACCTTAATCCAGCTTGTTACTTGCAATCCGCACATGTTACCTATGGCTGCCGTCGAGGAAAAAGGCAGGCCCTTTGATTCGAAATACATAGTCATGTGCACCAACAGGGAGTACGCTCACTCTGAGGCTGATCTGGCTGATCAAAATGCTTTTCTGAGACGACGAAAGCTGCTGTGGAAGGTTGCGAGAGATGATCGCGTGGCTTTCGACCCCACATGTTGTTGGAAAAATCTTCTATTTACTAGAATGGATTCTCTCCATCCACGCCGCCGGCACGTTGATCAGGTGACCCTGACTTTCCCAGACATGATTGCATATTCAGCAAACCAGGCTAGAGCACATTTTGCTCTAGAACAGACAATGTTGGACAGCATTTCGACCTGCACGGACTCCTTTCATCTTGAGGAGGACCAAAATGGCCACTTTCGAGTGGTTTTTGACAACGTGGATGCTGAGGTCCACAATGCGGAAATGAGGAGGGCCAATAATCCCAACATTGAGAGGTATAGAGTCCAGAGTGGAGAATCTTTCACTTATTTTAGTTGCCAAAGACCACTTAGTCAGGGACAACATTGCCCAAATTTTGCAAATCACGTGGACGAATTTAATTCATGTACTCTTGACACGATTGGGGGCAAAGTTTTCTGTTCTAACGGGGGGGTTTTCCCAAGTGAAAGTGTTCAGTGGTCCAGTCATGAAAGTTATTTCATTGACAATATGGCTAAGTCAACGGGCTTGTTCGAGGTACAAGTGAATTTGCTGGCCAGTTTATTGATGGACGACACAAATATGGAAGAATTCATGGACTGGAGAAGGAATGTGCTTTTTGACGATATAGTTGGAGATGGTATTGACCCCCCTGCTTCTATCCGCAGTAAGAATCCGGATGTTCCGGATACGGTGTTCCAAGAATATTGGTCCAAACTCAGCGATCGTTCTCGATACTTGGCCATTATGTGGCGAGATCTCACGCCGAAATCATCACTGAAACAAGCCATAGAAGGATTCAAGGAAGCAATCTCTGGGATTAAATGTGTACGGCTTTGGGAGTCATTGCCCTTTTGGCTTAAGTGGGCAATCGGATTTTTCGGCATCTTTGCGGGAGGATGTGTTGTCTATTCTTCTCTCAGGTGGCTCGCTGTACTTGGCCAACAGACTGGAGTTAAGCTGTTGTCCTATGTTTTAGGAGCAGATCCTTTCCAAGTACAGGGTACTTCATCAGGCGGTGATGAAAGGATCAACCGCACGCGACGTGTTGCTGTGCGAGGTTTCAGAGCACAGAGTGCTGCTTATGAGTCGATCCCGCATCCTGACGCATGGGACAAGTGCAAGAAAGCAATGATTCGAGTTGAGGGGACGGGCATCAAATCACACAGACCGTTTGCCTTCTGTGGTCTGATGATAGGTCCACGAAGATTCGTGGCCCCTGCTCATAGCGTTGTTCTGATGAACTTTGGTGTTTCTATGATGGTGACGAATGGTGAGGGCATGGGATGTACTTTCTATTGTAGATATGCACCCACCATGCTTGATGAATACCCAGAGTTTCCGGGAAAAATGAAAAGTTTGGTTGTGATGGAGTATTCAAGATTGACGCCACCTCTGGCTTATTACAGCAACATAGTAACTGATTTTTCCCTGGAGGTGAGTAGGTGTGTGCCGGGCTTTATCATGCCAAATATCAACCCTGACAGCGCTGATTTCCCACATGTCACCGAATTCTTTAAGGTGAATAGAGTTGACGACATTGCTGATGGAGCCTCAGGCATGACTTGGAAAGCTATTAAAACCTTCCATGCCAAGGAGGTCGGGAAAGATGGAATGTGTGGACGTCTCGCCCTAATAGACAGGAATAACACTCTGCAAGTGGTTGGAATGCATTGCTATGGGCGACCATCCGACTCGATTTTCTGTGACTTTGAAAAACATTTTGTTGTTGAAACTTATAAAGCACAATCTGAATATAATCAGTGTTTTGAGAGAACTCAGATAACTGAGATGGTGGATCTTGTTGGCACACTTGATGTTAGGGTTCCACGCTTAGAGAAAAGCCAGATTGAAAAATCCTTAATTCATGACACTTTAAAGGAAATGTGGCGTGACCCTCTAACTGAGCCTACAATTTTGTCTCGTGTGGATCCTCGCCCGCCCTATTCATATGATCCATACGAAATGGGAGTGAGGAAGTTTGATAAGGAGGCAGGACCATTTGATTTGAGAGAAGGGACAGAGTTCAACAAAGCCCTGGCTGATATCAAACAGAGTTGGTTGGATATTAAGCCGGAATCGTTTAAGATAGAACCGGTGTGTTCTTTGGATGTCGCCATTAATGGAGTCGATGGAATGCCCTATGCTGAAAATTTTCCGATTAGCACTTCAGAGGGATATCCTTATCTGCTCGAGCGCCAGCATGGAGAGAGTGGAAAATATAGATACTTTGAAGAGGATCTATCCGGAAAGAGGAAACCCAAGGGAGATTGGGTTAAGGACGTTGATGAGATTGAGAATCTGTGTGCTTCTGAAGAACTTGAAATTTTCTCCATCGCATGTGCAAAGGACGAGAAAACCAAATTGGCCAAGGTTTACGAGACTCCAAAAACACGAATTTTCGAAATCTTGCCCTTCACGTATAATCTATTGGTCAGAAAATATTTCCTGTTCTGGATGCAGTGGATGATGGAAAACCATATGAATCTTCCATGCAAGGTTGGTTTAGATTGTTTCTCCTATAACTGGGACATTATGGCATCTCAACATATGGCCTTTGCGAACCATTTTAATGGTGACTATTCGGGTTTTGACACGAATACGTCACGAGACATGATGATGAAAATTTGCGATATCATTTGCGATTTTGCTGATGATGGTGTTCGGAATCGCACTATTAGGAGGAATCTTATATGGGCTGCTGTGAACAGGAAATTGATAATTGGAGACAAGATTTTCGAAGTTAGAGGTGGCACCCCATCCGGCTTTGCTCTCACTGTGCTGATTAATTCTGTTATGAATGAATGGTTTTTGCGAGCGAGCTGGTATGCCATAATGCGTGTTCAGGAACCAATTCTGGCCAACAGTCGAGATTTTAGATCTCATGTGCGCCTTTCTGTTTATGGTGATGACAATGTTGTTTCCATGTCGAACCAGGTTGTCGAGCTGTACAATCTTGTAACCATTTCTGAATATCTCAAGCAATTTGGCATCAAATTGTCTGATGGTGCCAAAACTGGCGTACTTAAGAAGAGAATGAATTTTGAAGAGATCGATTTTCTCAAAAGAAAATGGACCGTTGGAAACTTTGGTTGGTTTCATTGTCCTCTTGATCGGACTTCTATCGAGGAACAATTGTTTTGGATAAAGAAATCAGATGACCCTCTGGCCAGCGTTCAGATGAATGTAGACAATGTTCTCCGTGAGTCATTTCATCATGGTAGAGACTATTTTGATACGATAAGAAGGGTCATCTCGAAAGCTGCTAATCTCAAAGGACTGGAAATTCTCCTGTTAAGTTTTGAAGATTGCGCAGACATGTGGACCACACAGAGAACTAAAGGCAATGCCAATGTTCCAGTGTGTTTCGATTTGCCTTACCTGAAGAAGCGCTATTTGGAGACCCAAGTGCGTGGCCTGGGCGAAAAGAGATTTGATCCGATTTATGGTATGACCATTGCGCGACTCAACAATTTTGATCCTAAATGGCGCCAAGCTGATAAGGTCGTGATTCTGACTGATGGGGGAGGTTCCTATGTTAAACACAATGGAACTTTGTGTTTAGATATCACTACTGATGGTGGTATGCAAATGAGAAAACTTCGAGAGTACATTTCCAATGGGGACTTTAGACGCTGCTATGTTACTCACCCCTCCAATGTTGCTGCTTGTGGGGTGTATGCACTTCTGACTTATCTCTTTGTGTTTCCTGAAGAAAAATCGATGTTGCATGCTTGGGTGGATTTGCTTGATGAAAATGCTTATAAGCAATTTATCAAACTTAATTCCTTCGTGTGAATATTGTGTGACTCTTGTGTCAGCTAGTGGTTAGGCTACTGTGTGTGATTTCCTAGCCACCATATTTAAATAAAATTGATGTGTGCTTATGTTTTTGAGTGTTTAGTGTGTGATGCTCTAAATTTATGCATGCTTTCTACC

**PSV-B_RNA2**

GCAAATCTAACCTGACCCTTTGGGGTGCCAAACAGGCTCTAGTAACTGCTATCTATGCATGATGTCCATGTGATCATGTCAATGGCCTCTCGATTCCATGGGACCCAGCATATATAGGAGGTATAAGCTTGCTGCAGGTAAATGCGATTCATGCAGCTTCTTCAAGTGGTTCTTTTTCCACTTTGTTCATTGGTGTTTTGGTCTTTCGGTCTTTAAGCCTTATAAGGAGGTTTTTGAGGATTATTATTATTCTGCCTTAAGAGCTTCAGATGCTCGTCTTCGAGTCAATTTGAAATTTAAGAAAACTCCTGCTTTCGACTTGCTTGTTCTATACGTTGGACACAACTATTTCCCTTGTAGATTCTGTTGGTTTCATCAACATTATCATACTATTTTCCGTTGCCAACTTAAATCTGTTATTATGTCTCAGACAAGGTCTGTGCAGGAGGTTTTCACTGGTGCTGGCCGGCAGCTTGGAACTAGTGCCTCTAATATAGCTCGAAGTTTGAGCCAGGTTGGTGATGAGACCATTCTCAATACTGCTTCTCCAATCGTAAAGATGTTTGATGAAGCTGTTAGAAACAAAAACTTTCATGTTCTTCCGAGGAGAGTCCAACAAATGAAAGATGTTCTTTTAGGAGTTTCTAAGAAATATGTCCATTTTGACATTGTCGATGCCACCACTAGCGCTGCTGTGGAGGACGCAGTCGTTGAAACTCCGATCCCTCTAGATCAATATGTTAATATTGATAAAGCTGAGGAATCATTGCGAAGTAAAGAGGAGAAGAAACTCGACATTGTTAATGGAAAAACATGTATTGTCGAGGCCATTGCTCTGCAGTCAACTTCTAATACTCCTGCAACAAGTGGTGGCGTTGTTTTGACCATGGCCGTTGATAGTAGGGCCAACAATCCTGAAGATGCGATTCTTGGTGGGCACATACATGTTGCCAGCCGTAGCGATACAGCTGGTAGCATGTTTATGCCATTTTTCAAGCTCAATACTAGAGATCCATATTTGTCAACCGCCCTTAAAATCATGACAATTAGTTCGGGATTTGACATGAAAGAGGGCTCCATAGTGGCAACTATCAAGCCACTGGTTGTTGGTGAAATAGTTCAAGACCCTCAATATACTACCATCAAGAAGGGTGTTTTGAAAGAGATTATGAAAGCTGGCCCTGTGACTCACACTGATCACTGCTTACCTATTCATCCGCGATTGCCCGAAGGACCACGCAGTACTGAGATTGTGGTACCATGGGAAAGTGCTACGCAGGCCTTGCTTTACTCGCACACTGCGGATGGAACAAGTGTATGGAAGAACGCTACAAAAGGAAAACAGAGCGTTATCTTTGAACTGCCGCGACATCTCACCAGTTCGCGGCGCATCAATCAAATAATCGCTGAAACCTCTGATCCAAATTTGCTTGGAAGGACTGATGTTAGAGAGGTTTCTGATAATTATACTGAACAGGGTTCCGGCGACAGAGAGGAACACATGCCAACTGAGGGCTCAGGGAGACAGGAGAAATTCGACGGCTTGCCTCATGCTTTTACGCCTGAGGAAAGCATAGTGAAGAAAGATGATGTCCCTTCGATGAAAGATAGGGGAGGAGAGTATGGTTCTACATACGTTTTGGAGGATGACAAAATTCTCTTCTCTGCACGCTATGTGGTGGACAACACAGCAGAGACAGGAGCCAGCCTGGTAACTGTCCAACTTATGGACGATGTTCTTTCGCGTGGGTTTGAGTCTGCTGTGGCACGATTGGCCCTTATCATGCCAATTGTGGAACCAATCATTAAAATAAGGGTTACTTACACCATACCCTCACTGTGTAGCGTGCCACTTATTCTTTCCTGGGACGAGTCTGGGGACAAACTAAAGAAAGCCGTTGTGCTTGAGCGAATCCTCAATCAGCCGTCCATAATAATGAATTCACACAGTGTTGTGACAAGTCATGAGCTTGTCGTACGACCAGCTGGTCACACAGGTAGGTTCAACTTATTCGCATCAGGAGCTGAACGAATGGGAGCTTTCCACATAGTTTCGTGTGGGCATAAGCTCAAAGGAGACGTTAAGGTTAATCTAGCTGTTGACATTCTGTTTTGCAAGAATACCATCATGATGCCATTGGCGCATCGACCTGATAGGCCCATTGCTAGCATACCAAACGCAACGTTCAAAATGCTAGAACGAGTTTCAATGGGTGATGTGAATTTGCACCATGTGCTATCACTACATAAATTCACGGATAAATCACCAACTGGTGATATTTATTTAATTTCGGTAGTACCTGGAATTTGTTCCATTTCTGATAAGGGGACCAAGTCATTCGTCAGCACATTTGGCAAGATGCTGACAATGTGGAATTTTTGGCGCGGGGACGCAATTATAGAAGTGTCATGCGCAGCCAGGAAAAGCATAGCTGGCTCATGCACTTTCTTTGTGGTGCCACCAGGGGTTGAAACTGCCCTGCTGTCGCCAACTATCCTCGCAGGTTTTCCTTTCTTCCGTGTCGATTTTTCCATGGATAAACCTGTCAAATTCAAATTCCCAGTCAATAGTTGGCTCAATTGGTGTGTAACGCAAGGAATTGATGACTTTAATGCTGTGGACACCAACAACTGTGAGAGTTCCCTCATTATGCGTCTAGATCAGGCTCCTTATGATAATATGGGGGATGCTGTGGAAGTGCTCATGTGCACACGCATAGTAGCAGTCAAGAATTTGGAAGTCTCTGAGAGAGCTTCCTGTGCAAAACAAAAAGGGGCAAATTTCGCTAAAACGTTTGATATGGGTCGAATCCTCCATGACCAAGTGCTGACTGCTTATAGAGCACAATCTGAAGTCCCCGGATTTCAAAATGAGGGCTTTGCGCAAAGTTTGGTCTTTTACCCTCTCGAAGTTGCTGAGAATGGAGAGGGTGCTCGTTCAGTTATGTTGTCATTCCCTGTTTGTTTCAACTATCGGGATGACATGTTTGAGAGGAAAGGTCAAGGGAAAGCTGGACTCGTTTACATTGACCATTTGAATCCTTACCACCAACTATTTTCAGGGGTCTCCTATTACTCGTGCGGCATTGAAGTGCTCATCTATGTAGAACACGAGGATGGCTCCTCAGGCAAGGCCATTGCCGTTTTCAAAAACGGCTCAATGGAAAATCATGTTTATGGCCAGACCACTGCAACAAATGATGGAAATTATGGTGGCGGAGTGTCAATGGACTATATGTCAGCAAGTGGTGCTCTGTACCTTAGGATTGAACCACGCAACTTCAATTGCAGGGGACGCGTGAGAACGAAACAAAGTCACAGATTCCTTGACACTTATGGGGTTTGTCACGTCAGTGTACCTCCTTTTGATGTGGTCAAATGCATCAGAGTTTTCACTCGACCATTCGGGAAGATATCCATTTATGGTGTGCGAACTCCCGAGATGGATGTAGATGCTAATTCAGGGAAGAGAAGATGCTCATGCGTCTATCTCACTAATTCTGATGCAAATGTTGGAGGGTACTAGAGCTGCGATGATTTGACAAGTGGTTGCGACAGCAACAACTGCAATAAAGACTGAATGATTTGCTCCTTTCTTCAGTCACGTGTTATCTGCTTCTTTATCATTATTATTGTGCTTCTTGGATGATGTTATTTCGTTGTGCGTGTGCTTGCTGCTTGTTGTTTCTTTGCAACTATAATGGTTGTGGACTCTTGTCCATCCATGAATAATTATTATTATTATTGGTGTGTATTTAGTTTTGAAATGTTTGTGTGTGATGTTTTAAATCTATGTATGCTTCCTAAC
